# mTOR signaling is activated by FLT3 kinase and promotes survival of *FLT3-*mutated acute myeloid leukemia cells

**DOI:** 10.1186/1476-4598-9-292

**Published:** 2010-11-10

**Authors:** Weina Chen, Elias Drakos, Ioannis Grammatikakis, Ellen J Schlette, Jiang Li, Vasiliki Leventaki, Efi Staikou-Drakopoulou, Efstratios Patsouris, Panayiotis Panayiotidis, L Jeffrey Medeiros, George Z Rassidakis

**Affiliations:** 1 Department of Hematopathology, The University of Texas M.D. Anderson Cancer Center, 1515 Holcombe Blvd., Houston, Texas 77030, USA; 2 First Department of Pathology, National and Kapodistrian University of Athens, School of Medicine, 75 Mikras Asias str., Athens, 11527, Greece; 3 Department of Hematology, National and Kapodistrian University of Athens, Laiko General Hospital, 17 Agiou Thoma str., Athens, 11527 Greece

## Abstract

Activating mutations of the *FLT3 *gene mediate leukemogenesis, at least in part, through activation of PI3K/AKT. The mammalian target of rapamycin (mTOR)-Raptor signaling pathway is known to act downstream of AKT. Here we show that the mTOR effectors, 4EBP1, p70S6K and rpS6, are highly activated in cultured and primary *FLT3*-mutated acute myeloid leukemia (AML) cells. Introduction of *FLT3-ITD *expressing constitutively activated FLT3 kinase further activates mTOR and its downstream effectors in BaF3 cells. We also found that mTOR signaling contributes to tumor cell survival, as demonstrated by pharmacologic inhibition of PI3K/AKT/mTOR, or total silencing of the *mTOR *gene. Furthermore, inhibition of FLT3 kinase results in downregulation of mTOR signaling associated with decreased survival of *FLT3*-mutated AML cells. These findings suggest that mTOR signaling operates downstream of activated FLT3 kinase thus contributing to tumor cell survival, and may represent a promising therapeutic target for AML patients with mutated-*FLT3*.

## Background

The *FMS*-like tyrosine kinase-3 (*FLT3*) receptor, also known as CD135, is a tyrosine kinase type III normally involved in hematopoietic progenitor cell proliferation, survival, and differentiation. Previous studies have shown that constitutive activation of FLT-3 is involved in leukemogenesis, partially through phosphorylation/activation of the serine-threonine kinase AKT, which occurs in a substantial subset of acute myeloid leukemia (AML) cases [1,2]. A frequent mechanism of FLT3 activation is mutation of the FLT3 gene, either internal tandem duplication (ITD) within the juxtamembrane domain (FLT3-ITD), or point mutations within the activation loop of the second tyrosine kinase domain (FLT3-D835/836). FLT3 gene mutations are found in approximately one third of AML patients, and are associated with inferior prognosis [3].

The mammalian target of rapamycin (mTOR), an important downstream effector of AKT, is a master regulator of cell growth and metabolism [4]. There are two mTOR multi-protein complexes, mTOR-Raptor/mTORC1 and mTOR-Rictor/mTORC2. mTOR-Raptor/mTORC1 is sensitive to the natural macrolide rapamycin and regulates the rate of protein translation [4,5]. This regulation is accomplished, partially, through phosphorylation of the ribsosomal protein S6 kinase (p70S6K) and subsequent phosphorylation of the ribosomal protein S6 (rpS6), or phosphorylation and inactivation of the eukaryotic initiation factor 4E (eIF4E)-binding protein-1 (4E-BP1), dissociating 4E-BP1 from the RNA cap-binding protein eIF4E, thus promoting cap-dependent translation of mRNAs [4,5]. mTOR-Rictor/mTORC2, usually insensitive to rapamycin, phosphorylates AKT at serine residue 473, contributing to AKT activation, and establishing an autoregulatory loop [6].

Recent studies have shown that rapamycin, and its analogs, have substantial antitumor activity in hematologic malignancies, including AML [7,8]. However, the significance of *FLT3 *gene mutation in the activation of the mTOR pathway is not clear. In this study we hypothezised that the mTOR signaling pathway has an oncogenic role in *FLT3*-mutated AML cells. We show that mTOR signaling is highly activated in *FLT3*-mutated AML cell lines and primary cells. We also demonstrate that total inhibition of mTOR signaling results in cell death, specifically of *FLT3*-mutated AML cells, whereas inhibition of the FLT3 kinase results in downregulation of mTOR signaling. Our findings suggest that mTOR signaling operates downstream of mutated FLT3 kinase and that AML patients harboring *FLT-3-*mutations may benefit from experimental therapies that target mTOR signaling.

## Results and Discussion

### The mTOR signaling pathway is activated in *FLT3*-mutated AML cells

**Figure 1:**
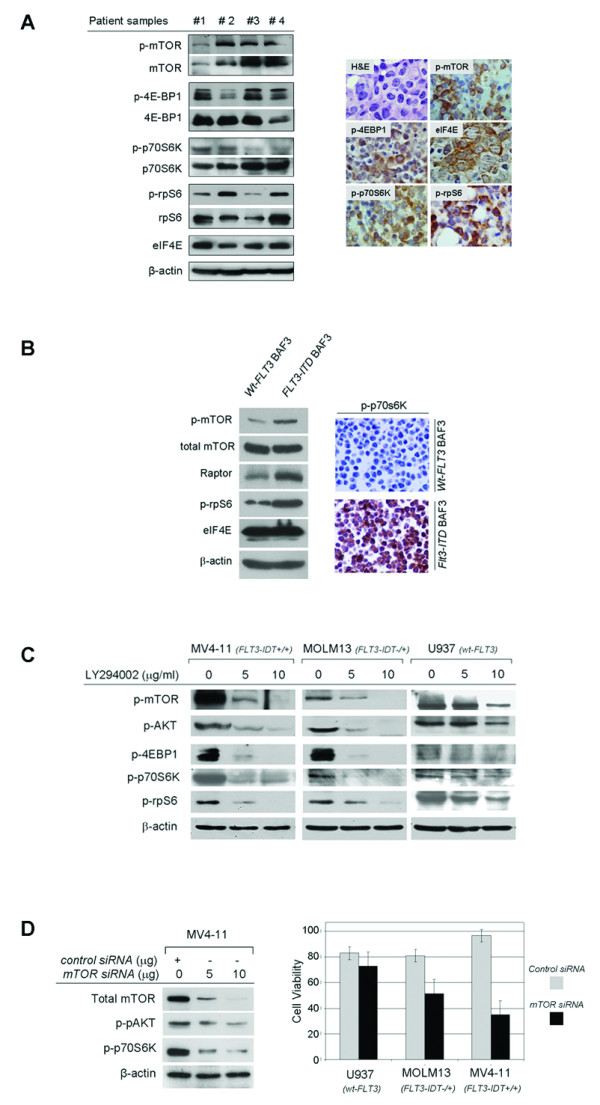
**mTOR signaling pathway is activated and contributes to the survival of AML cells harboring mutated FLT-3**. **A**. Western blot analysis of primary AML cells with mutated *FLT3-*ITD showed activation (phosphorylation) of mTOR kinase and downstream mTOR mediators (*left panel*). These data were confirmed also with immunohistochemistry on *FLT3*-mutated AML bone marrow samples (*right panel*). In positive cases, the majority of tumor cells show expression of phosphorylated (activated) mTOR, p70S6K, rpS6 and 4EBP1 with a cytoplasmic staining pattern (DAB chromogen, hematoxylin & eosin [H&E] counterstain, original magnification ×400). **B**. A causal association between activation status of mTOR signaling pathway and activating mutations of *FLT3 *was further supported by the upregulation of mTOR, Raptor as well as phosphorylation (activation) of downstream effectors such as p70S6K and rpS6 proteins in BaF3 cells stably transfected with mutated *FLT3 *compared with BaF3 cells transfected with *Wt-FLT3*, as shown by Western blot analysis (*left panel*), or immunohistochemistry performed in cell blocks (*right panel*). **C**. Western blot analysis of AML cell lines showed that pharmacologic inhibition of PI3K and mTOR kinases by LY294002 resulted in downregulation of p-AKT and downstream mediators of the mTOR pathway. This effect was more pronounced in the MV4-11 and MOLM13 cell lines harboring mutated *FLT3 *comparing with the U937 cell line with Wt-*FLT3*. **D**. Silencing of the *mTOR *gene by transient transfection of *mTOR*-specific siRNA resulted in downregulation of mTOR and p-AKT signaling (*left panel*), associated with decreased survival of *FLT3-*mutated MOLM13 and MV4-11 cells by 30% and 62%, respectively, as compared with a small decrease by 13% of the Wt-FLT3 U937 cells (p < 0.05), 48 hours after treatment (*right panel*).

Initially, we investigated the mTOR pathway activation status by immunohistochemistry and Western blot analysis of bone marrow samples and primary peripheral blood AML cells, respectively, in patients harboring *FLT3 *mutations. Imunohistochemical analysis showed that 10/12 (83%) p-mTOR, 10/13 (77%) p4E-BP1, 13/14 (93%) p-p70S6K, and 11/12 (83%) p-rpS6 indicating mTOR activation. All AML tumors (n = 6) with dual mutations (FLT3-ITD and tyrosine kinase point mutations) were positive for p-mTOR, p-p70S6K, and p-rpS6. Also, all AML tumors (n = 15) expressed high levels of eIF4E (Figure 1A). This is the first immunohistochemistry study of mTOR signaling proteins in AML. We also showed activated mTOR signaling by Western blot analysis (Figure 1), in agreement with others [9]. To further investigate a possible causal association between *FLT3 *mutation and the mTOR activation status, we used stably transfected *FLT3-*mutant BaF3 cells. Introduction of *FLT3-ITD *in BaF3 cells resulted in activation of mTOR signaling (Figure 1).

### mTOR Signaling contributes to FLT3-mutated AML cell survival

We assessed the role of mTOR signaling in the regulation of cell growth and survival of AML cell lines. Most previous studies investigating this issue were based on rapamycin treatment of AML cells resulting frequently in conflicting conclusions [10,11]. However, recently it was realized that rapamycin not only can indirectly and unpredictably inhibit the rapamycin-insensitive mTORC2 after prolonged treatment, but also frequently inhibits partially and not totally mTORC1, which is regarded as the specific target of rapamycin. For this reason, we employed the pharmacologic PI3K inhibitor LY294002, known to inhibit both the PI3K and mTOR kinase activity with the same kinetics, or total silencing of the *mTOR *gene using an *mTOR*-specific siRNA [12,13].

As shown in the Figure 1, treatment with LY294002 resulted in a dose-dependent decrease in phosphorylation of AKT, mTOR, 4E-BP1, p70S6K, and rpS6, associated with reduced cell viability due to increased apoptosis (additional file 1, Figure S1). Although changes in the levels of mTOR signaling proteins are similar in both FLT3-mutated AML cell lines, it seems that MV4-11 cell viability depends more on mTOR signaling (Figure 1, D*right*, and additional file 1, Figure S1) and this difference is also observed after combined inhibition of mTOR with rapamycin and FLT3 with GTP14564 (additional file 2, Figure S2). The biologic explanation for this finding is uncertain. The latter biologic effects were linked to downregulation of anti-apoptotic proteins involved in both extrinsic and intrinsic (mitochondrial) apoptotic pathways (additional file 1, Figure S1). In addition, transient transfection with *mTOR*-specific siRNA resulted in decreased phosphorylation/activation of 4EBP1, p70S6K, and rpS6 in a concentration-dependent manner (Figure 1). Of note, *mTOR *silencing was associated with decreased levels of p-AKT indicating that mTOR/Rictor complex may contribute to AKT activation in this cell system. Silencing of *mTOR *also resulted in decreased viability of the AML cell lines, MV4-11 and MOLM13 (both mutated-*FLT3*) as opposed to Wt-*FLT3 *U937 cells which were unresponsive (Figure 1).

Taken together, our findings from *mTOR *gene silencing and pharmacologic studies suggest that mTOR signaling may contribute to the survival of AML cells harboring *FLT3 *mutations, and are concordant with the results of other studies [8,14,15].

### mTOR signaling acts downstream of mutated-FLT3 Kinase

**Figure 2:**
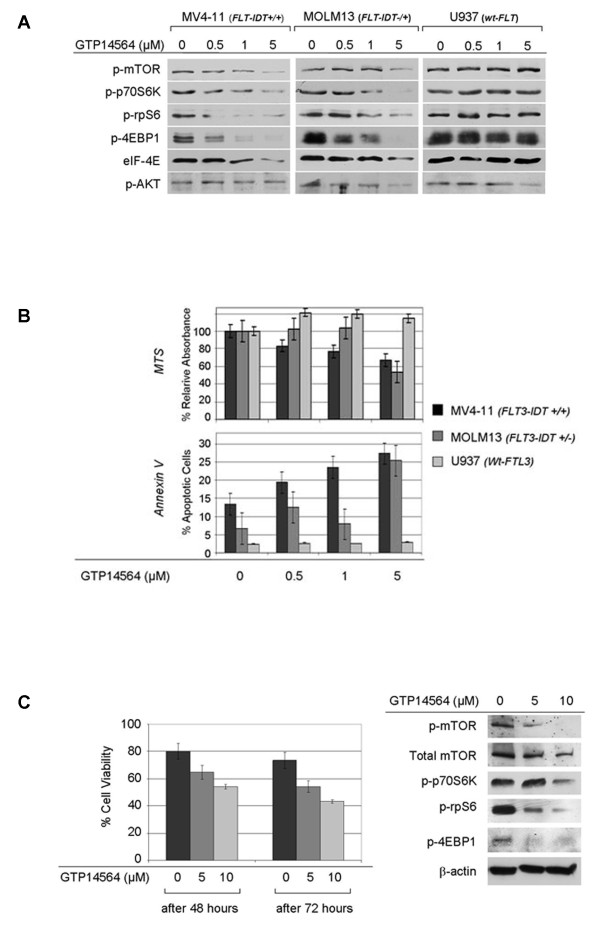
**Inhibition of FLT-3 results in downregulation of mTOR signaling, accompanied by decreased survival of AML cells harboring mutated FLT-3**. **A**. Pharmacologic inhibition by the FLT3-specific inhibitor GTP14564 resulted in a concentration-dependent downregulation of downstream mTOR mediators in the *FLT3*-mutated AML cell lines MV4-11, and MOLM13, with no effect on the Wt-*FLT3 *U937 cell line, 48 hours after treatment. However, note that pAKT is minimally affected in all three cell lines. **B**. Treatment of homozygously mutated-*FLT3 *MV4-11 cells with increasing concentrations of GTP14564 (up to 5 uM) resulted in dose-dependent decrease in cell proliferation, up to 33%, and apoptotic cell death was increased, up to 15%. Concentrations up to 1 uM had no significant effect on survival and the proliferation of the heterozygously *FLT3*-mutated MOLM13 cells. However, after treatment with 5 uM GTP14564, the proliferation of MOLM13 cells was decreased, up to 44%, and apoptotic cell death was increased, up to 19%. GTP14564 had no significant antitumor activity against Wt-*FLT3 *U937 cells (p < 0.05). **C**. Pharmacologic inhibition of FLT-3 by increasing concentrations of GTP14564 (up to 5 uM) resulted in a concentration-dependent decrease of cell viability, up to 31% (after 72 hours) (left panel, p < 0.05), accompanied by downregulation of mTOR signaling, as shown by Western blot analysis (right panel) in *FLT3*-mutated primary AML cells.

**Figure 3:**
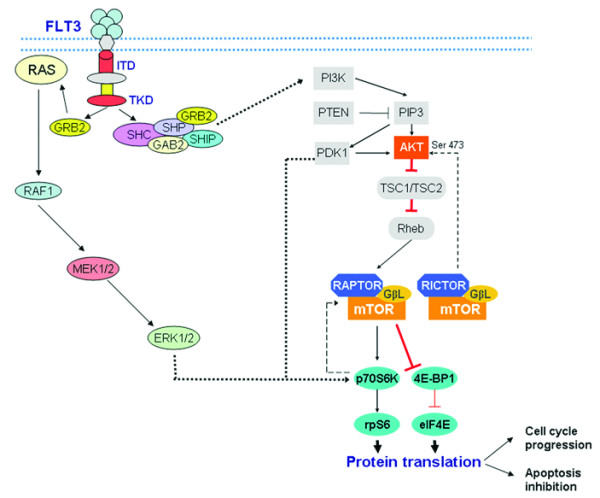
**FLT3/mTOR signaling in AML**.

To further test this hypothesis, we evaluated the selective *FLT3 *inhibitor, GTP-14564, on mTOR signaling in AML cells. No changes in activation of mTOR signaling proteins, as well cell proliferation or death were observed in Wt-*FLT3 *U937 cells after treatment (Figure 2). However, treatment of the *FLT3*-mutated AML cell lines MV4-11 and MOLM13 resulted in a concentration-dependent decrease of p-mTOR, p-p70S6K, p-rpS6 and p-4EBP1. These changes were slightly more pronounced in the homozygously mutated MV4-11 compared with the heterozygously mutated MOML13 (Figure 2). Unexpectedly, AKT (p-AKT) activation was affected minimally or not at all (Figure 2). The biologic explanation for this finding is uncertain, however, the possibility that activation of the mTOR signaling pathway induced by mutated FLT3 kinase may partly involve AKT-independent mechanisms cannot be excluded. These changes were accompanied by a proportional decrease in cell proliferation, and increase in cell death (Figure 2). These biologic effects were further augmented when combined treatment with GTP-14564 and Rapamycin was used (additional file 2, Figure S2). Downregulation of mTOR signaling accompanied by similar biologic effects were also observed after GTP-14564 treatment of *FLT3*-mutated primary AML cells (Figure 2). These data provide evidence that mTOR signaling is an important downstream mediator of the oncogenic pathway activated by mutated FLT3 kinase (Figure 3), and that direct inhibition of mTOR may possibly augment the therapeutic potential of FLT3 inhibitors[16]. Our findings are in agreement with previous observations that rapamycin in combination with the *FLT3 *inhibitor, PKC-412, markedly inhibits cell proliferation in murine BaF-*FLT3*-ITD cells and AML samples bearing *FLT3 *gene dual mutations of ITD and point mutation types, and that rapamycin inhibits cell proliferation in murine 32D-*FLT3*-ITD cells [2,8,17].

## Conclusions

In conclusion, our results taken together suggest that *FLT3 *mutations may lead to activation of mTOR signaling pathway and thus contribute to tumor cell survival and growth in AML (Figure 3). Therefore, selective inhibition of mTOR signaling pathway seems to be a promising therapeutic target for patients with *FLT3-*mutated AML.

## Methods

### Cells and Reagents

Three human AML cell lines were used; MOLM-13 (heterozygous for *FLT3-*ITD), MV4-11 (homozygous for *FLT3-*ITD) [18], U937 (wild-type *FLT3*), and murine BaF3 cells transfected with wild-type (Wt) *FLT3 *(BaF3/FLT3) or mutated *FLT3 *(*FLT3/ITD *and *FLT3/D835G*) were cultured under standard conditions as previously described [18]. The murine cell line BaF3 was kindly provided by Dr. M Andreeff (M.D. Anderson Cancer Center, Houston, TX). All four cell lines (MOLM-13, MV4-11, U937, BaF3) were tested negative for mycoplasma by standard polymerase chain reaction (PCR) methods. Ficoll-purified (Sigma Chemical, St Louis, MO) peripheral blood mononuclear cells from 4 AML patients having >60% blasts in blood and harboring *FLT3*-ITD mutations were also used for *in vitro *studies. The PI3K inhibitor LY294002, the mTOR inhibitor rapamycin and the FLT3 inhibitor GTP-14564 (all from Calbiochem. San Diego, CA) were applied at different concentrations and durations of time as indicated. The kinase inhibition experiments were performed at least three times with reproducible data.

### Immunohistochemistry

Fifteen formalin-fixed, paraffin-embedded, AML bone marrow samples (9 harboring *FLT3*-ITD mutation, and 6 harboring dual, *FLT3*-ITD and point mutations) were used for the construction of a tissue microarray. Immunohistochemistry was performed using histologic sections of the tissue microarray or cell blocks, and antibodies specific for p-mTOR, p-p70S6K, p-rpS6, p-4E-BP1, and eIF4E (Cell Signaling Technology) as previously described [19]. AML cases were considered immunohistochemically positive for any of the proteins analyzed if at least 10% of tumor cells showed expression after examination of 10 representative high-power fields (magnification ×400). The study was approved by The University of Texas M.D. Anderson Cancer Center institutional review board.

### Western Blot Analysis

Western blot analysis was performed as previously described[19]. The antibodies used included ^Ser473^pAKT (p-pAKT), total Akt, p-mTOR, p-p70S6K, p70S6K, p-rpS6, rpS6, p4E-BP1, 4E-BP1, eIF4E (Cell Signaling Technology, Beverly, MA), and β-actin (Sigma, St. Louis, MO).

### Silencing of mTOR by siRNA

The sequences of *mTOR *small interfering RNA (siRNA) (5'-GGAGUCUACUCGCUUCUAUTT-3' [sense]; 5'-AUAGAAGCGAGUAGACUCCTC-3' [antisense] and a negative control siRNA were purchased from Ambion, Inc, (Austin, TX). Transient transfection was performed by electroporation using nucleofector system (Amaxa Biosystems, Gaithersburg, MD), according to the manufacturer's instructions. Gene silencing experiments were performed twice with reproducible results.

### Cell Death, Apoptosis and Proliferation Studies

Cell viability was evaluated by trypan blue exclusion cell assay in triplicate. Annexin-V staining (BD Biosciences Pharmingen, San Diego, CA), detected by flow cytometry, was used to assess apoptosis, as previously described [20].

Proliferation of the cells was assessed by using a tetrazolium compound [3-(4,5-dimethylthiazol-2-yl)-5-(3-carboxymethoxyphenyl)-2-(4-sulfophenyl)-2H-tetrazolium, MTS] (Promega, Madison, WI, USA), as previously described [20]. All experiments were performed at least twice.

### Statistical analysis

Data for cell viability, growth and apoptosis are shown in diagrams as mean +/- SD and their differences between different cell lines and concentrations were evaluated using Student t-test. p-values < 0.05 were considered statistically significant.

## Competing interests

The authors declare that they have no competing interests.

## Acknowledgements

This study was funded by the NIH Joe Moakley Leukemia SPORE grant to The University of Texas M.D. Anderson Cancer Center. G.Z. Rassidakis is a recipient of a Leukemia SPORE Developmental Research Award.

## Authors' contributions

WC designed and interpreted experiments and contributed to the writing of the manuscript, ED interpreted experiments and contributed to the writing of the manuscript, IG executed experiments, EJS contributed vital reagents and contributed to the writing of the manuscript, JL, VL and ESD executed experiments, PP and EP contributed vital reagents and contributed to the writing of the manuscript, LJM interpreted experiments and contributed to the writing of the manuscript, and GZR designed and interpreted experiments and contributed to the writing of the manuscript. All authors have read and approved the final manuscript.

## Supplementary Material

Additional file 1 **Inhibition of the AKT-mTOR signaling pathway results in apoptotic cell death of AML cells harboring mutated FLT-3 (Figure S1)**. **A**. Pharmacologic inhibition of the *AKT-mTOR *signaling pathway by using 10 μg/ml LY249002 resulted in decreased cell viability and apoptotic cell death by 32% and 31% respectively in MOLM13 cells harboring heterozygously mutated *FLT3 *and by 48% and 75% respectively in MV4-11 cells harboring homozygously mutated FLT3, 48 hours after treatment (p < 0.05) (Figure S1A). **B**. Western blot analysis showed that LY249002-mediated cell death was accompanied by downregulation of the inhibitor of the extrinsic apoptotic pathway FLIP_S/L _and the inhibitor of the intrinsic apoptotic pathway BCL-XL in both MOLM13 and MV4-11 cells (Figure S1B). Click here for file

Additional file 2 **Combined treatment of AML cells with a FLT-3 inhibitor and a small dose of rapamycin results in enhanced cytotoxicity specifically in AML cells harboring mutated FLT-3 (Figure S2)**. Combination of 5 nM GTP14564 with 1 nM rapamycin resulted in enhanced apoptotic cell death, by 28% and 63%, in MOLM13 and MV4-11 cells harboring heterozygously and homozygously mutated *FLT3 *respectively (p < 0.05) (Figure S2), as compared to 19% and 15% induced by 5 nM GTP14564 alone (Figure 2). No effect was observed in U937 cells harboring wt-*FLT3*. Click here for file
