# Supplementary figures and images for: mTOR signaling is activated by FLT3 kinase and promotes survival of FLT3-mutated acute myeloid leukemia cells
Source: Mol Cancer. 2010 Nov 10;9:292. doi: 10.1186/1476-4598-9-292 (PMC2993677; doi:10.1186/1476-4598-9-292)

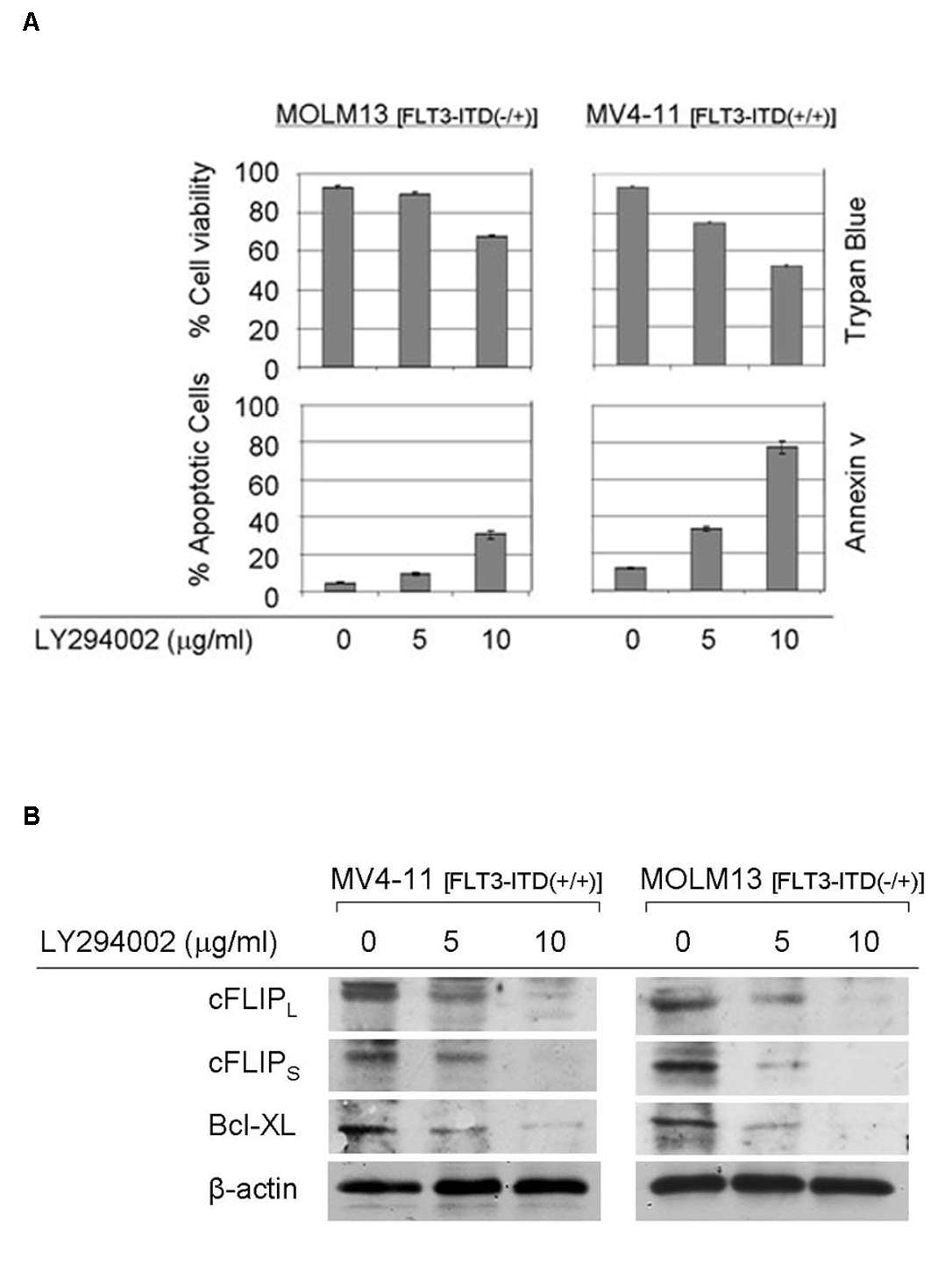

Supplement: Additional file 1 — Inhibition of the AKT-mTOR signaling pathway results in apoptotic cell death of AML cells harboring mutated FLT-3 (Figure S1). A. Pharmacologic inhibition of the AKT-mTOR signaling pathway by using 10 μg/ml LY249002 resulted in decreased cell viability and apoptotic cell death by 32% and 31% respectively in MOLM13 cells harboring heterozygously mutated FLT3 and by 48% and 75% respectively in MV4-11 cells harboring homozygously mutated FLT3, 48 hours after treatment (p < 0.05) (Figure S1A). B. Western blot analysis showed that LY249002-mediated cell death was accompanied by downregulation of the inhibitor of the extrinsic apoptotic pathway FLIPS/L and the inhibitor of the intrinsic apoptotic pathway BCL-XL in both MOLM13 and MV4-11 cells (Figure S1B). [file 1476-4598-9-292-S1.TIFF]

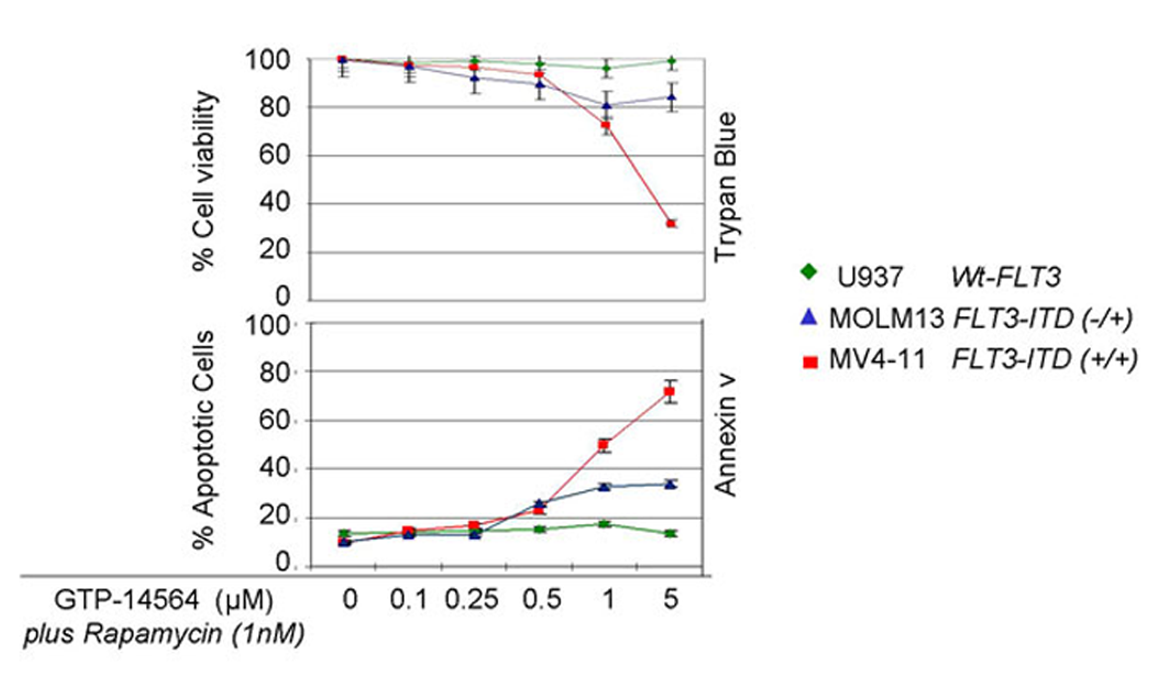

Supplement: Additional file 2 — Combined treatment of AML cells with a FLT-3 inhibitor and a small dose of rapamycin results in enhanced cytotoxicity specifically in AML cells harboring mutated FLT-3 (Figure S2). Combination of 5 nM GTP14564 with 1 nM rapamycin resulted in enhanced apoptotic cell death, by 28% and 63%, in MOLM13 and MV4-11 cells harboring heterozygously and homozygously mutated FLT3 respectively (p < 0.05) (Figure S2), as compared to 19% and 15% induced by 5 nM GTP14564 alone (Figure 2). No effect was observed in U937 cells harboring wt-FLT3. [file 1476-4598-9-292-S2.TIFF]
